# The XVI International Conference on AIDS: The place to be!

**DOI:** 10.1186/1742-4690-3-9

**Published:** 2006-02-03

**Authors:** Mark A Wainberg

**Affiliations:** 1 McGill University AIDS Centre, Jewish General Hospital, Montreal, H3T 1E2, Canada

## Abstract

This editorial represents a plea to retrovirologists to attend the XVI International Conference on AIDS that will take place in Toronto, Canada between August 13–18, 2006. In short, it is vital that politicians and opinion leaders understand that basic scientists are no less committed to the worldwide battle against HIV than community activists or non-basic scientists, and the best way to demonstrate this is by showing up.

The XVIth International Conference on AIDS will take place in Toronto, Canada between Aug. 13–18, 2006. It is vital that this meeting be attended by basic retrovirologists working in the field of HIV as well as by clinicians, epidemiologists and social scientists concerned with preventive and other aspects of HIV research. The reasons for this are simple. The International Conference on AIDS is the most media-intensive event in the world in regard to dissemination of information about the HIV epidemic. In addition, the International Conference on AIDS is the only one of its type that attempts to bring together scientists working on all aspects of the HIV epidemic together with members of industry, community representatives, and their supporters. As such, this is probably the only conference in the world that attempts to appeal to a broad spectrum of attendees and, in a sense, attempts to be "all things to all people". Although such a lofty objective can never be completely achieved, the efforts involved in trying are both noble and worthwhile.

Quite simply, the International Conference on AIDS probably does more to promote HIV awareness and education throughout the world than any other conference or group of scientific papers can currently achieve. In order to sustain this level of interest and support, it is vital that the conference attracts a critical mass of compelling papers that can articulate hope for the future in regard to an improved understanding of the HIV replication cycle, development of novel targets for drug development, and more appropriate use of both current and future drugs in a therapeutic setting. Moreover, these considerations apply as much in the context of developing countries as they do in countries such as the United States, Canada, and the nations of Western Europe. For example, it will be important to discuss the initial results of the PEPFAR program of the US government for purchase and distribution of antiretroviral drugs in Africa, which currently reaches at least 400,000 people. The latter is by far the most generous program of its type in the world and it is appropriate that the Conference make it a focal point in regard to expanded access to these life-saving drugs.

Scientists throughout the world who work on HIV should consider it their obligation to submit abstracts to the International Conference and to attend. We, who work in this field, have been the beneficiaries of major grant support as a result of increased levels of funding that are now available in the field. These increased levels of funding have come about, in part, because of political pressure brought to bear by community groups who have lobbied for the accomplishment of research that will make a difference. In addition, there are obvious political and economic factors that have also resulted in increased levels of funding in recent years. Major philanthropic organizations, such as the Gates and Soros Foundations, etc have also channeled more money into HIV research because of the global catastrophic situation.

Against this background, it is important that all scientists show solidarity in regard to the HIV crisis. It is not sufficient for any of us to want to work on problems that relate to the ideas in our grant applications without considering the global dimensions of the pandemic at the same time. The Toronto Conference organizers hope that we will attract a large number of first-rate abstracts that will make ours a first-rate scientific conference. Of course, we also want to organize a conference that will result in antiretroviral drugs becoming available to the millions of people around the world who are so desperately at need. Basic scientists as well as others in the field can vote with their feet by coming to Toronto and ensuring that the Conference is a major success.

All of this recognizes that basic scientists often derive more direct benefit by attending specialized meetings. The International Conferences on AIDS cannot compete in this regard. Nevertheless, it seems also important for basic scientists to interact with other disciplines and to meet industry as well as community representatives. The International Conference on AIDS provides the best podium for this.

We look forward to welcoming the readers of *Retrovirology *to Toronto for what will be a stimulating and challenging conference that will provide specific information to those who attend specialty sessions as well as more general information to those who want to learn how they personally can play a role in the global fight against HIV/AIDS.

